# Impact of Valve Size on Paravalvular Leak and Valve Hemodynamics in Patients With Borderline Size Aortic Valve Annulus

**DOI:** 10.3389/fcvm.2022.847259

**Published:** 2022-03-09

**Authors:** Yeela Talmor-Barkan, Ran Kornowski, Noam Bar, Jeremy Ben-Shoshan, Hanna Vaknin-Assa, Ashraf Hamdan, Boris Kruchin, Israel M. Barbash, Haim Danenberg, Gidon Y. Perlman, Maayan Konigstein, Ariel Finkelstein, Arie Steinvil, Ilan Merdler, Amit Segev, Alon Barsheshet, Pablo Codner

**Affiliations:** ^1^Rabin Medical Center, Sackler Faculty of Medicine, Tel-Aviv University, Tel-Aviv, Israel; ^2^Department of Computer Science and Applied Mathematics, Weizmann Institute of Science, Rehovot, Israel; ^3^Tel-Aviv Medical Center, Sackler Faculty of Medicine, Tel-Aviv University, Tel-Aviv, Israel; ^4^Chaim Sheba Medical Center, Sackler Faculty of Medicine, Tel-Aviv University, Tel Aviv, Israel; ^5^Hadassah Medical Center, Hebrew University, Jerusalem, Israel

**Keywords:** borderline aortic annulus, transcatheter aortic valve implantation, paravalvular leak, valve hemodynamics, multi-detector computed tomography

## Abstract

**Background:**

Transcatheter heart valve (THV) selection for transcatheter aortic valve implantation (TAVI) is crucial to achieve procedural success. Borderline aortic annulus size (BAAS), which allows a choice between two consecutive valve sizes, is a common challenge during device selection. In the present study, we evaluated TAVI outcomes in patients with BAAS according to THV size selection.

**Methods:**

We performed a retrospective study including patients with severe aortic stenosis (AS) and BAAS, measured by multi-detector computed tomography (MDCT), undergoing TAVI with self-expandable (SE) or balloon-expandable (BE) THV from the Israeli multi-center TAVI registry. The aim was to evaluate outcomes of TAVI, mainly paravalvular leak (PVL) and valve hemodynamics, in patients with BAAS (based on MDCT) according to THV sizing selection in between 2 valve sizes. In addition, to investigate the benefit of shifting between different THV types (BE and SE) to avoid valve size selection in BAAS.

**Results:**

Out of 2,352 patients with MDCT measurements, 598 patients with BAAS as defined for at least one THV type were included in the study. In BAAS patients treated with SE-THV, larger THV selection was associated with lower rate of PVL, compared to smaller THV (45.3 vs. 64.5%; pv = 0.0038). Regarding BE-THV, larger valve selection was associated with lower post-procedural transvalvular gradients compared to smaller THV (mean gradient: 9.9 ± 3.7 vs. 12.5 ± 7.2 mmHg; *p* = 0.019). Of note, rates of mortality, left bundle branch block, permanent pacemaker implantation, stroke, annular rupture, and/or coronary occlusion did not differ between groups.

**Conclusion:**

BAAS is common among patients undergoing TAVI. Selection of a larger THV in these patients is associated with lower rates of PVL and optimized THV hemodynamics with no effect on procedural complications. Additionally, shift from borderline THV to non-borderline THV modified both THV hemodynamics and post-dilatation rates.

## Introduction

Aortic valve stenosis (AS) is the most common valvular heart disease among elderly (1). Transcatheter aortic valve implantation (TAVI) has become an established and effective therapeutic procedure for symptomatic patients with severe AS regardless of procedural risk (2, 3), and recently is offered to a younger and lower risk population. These changes in TAVI candidates emphasize the need for optimal transcatheter heart valve (THV) implantation to achieve procedural success and prolonged durability. The selection of an appropriately sized THV is a crucial component of the TAVI procedure. Valve undersizing may lead to paravalvular leak (PVL), valve embolization and poor hemodynamics. Oversizing may result in coronary occlusion, atrioventricular block, mitral valve injury, periaortic hematoma, septal or annular rupture (4).

Multi-detector computed tomography (MDCT) is the gold standard method for pre-procedural planning and annular sizing of both balloon-expandable (BE) and self-expanding (SE) THV (5). THVs are currently available in a limited number of sizes and the manufacturer's sizing guidelines allow for a gray area with considerable overlap, where patients with borderline aortic annulus size (BAAS) may be candidates for either of the two suitable THV sizes (smaller or larger). Recently, study from the PARTNER 3 (Placement of Aortic Transcatheter Valves 3) trial demonstrated that in selected patients with annular dimensions in between 2 valve sizes, the larger THV device oversized to both the annular area and perimeter reduced PVL and optimized THV hemodynamics (6). Meanwhile, BAAS remains a common challenge during device size selection and the most effective THV selection strategy for these patients remains unclear.

The aim of the present study was to evaluate outcomes of TAVI, mainly PVL and valve hemodynamics, in patients with BAAS (based on MDCT) according to THV sizing selection in between 2 valve sizes. In addition, to investigate the benefit of shifting between different THV types (BE and SE) to avoid valve size selection in BAAS.

## Methods

### Study Design and Methodology

We performed a retrospective analysis from the Israeli multi-center TAVI registry, including patients with severe symptomatic AS and BAAS, measured by MDCT, undergoing TAVI with SE-THV (CoreValve, Evolut R and Evolut PRO) or BE-THV (Sapien XT, Sapien 3) during the years 2015–2019, at 1 of 4 tertiary centers in Israel: Rabin Medical Center, Sheba Medical Center, Tel-Aviv Sourasky Medical Center, and Hadassah Medical Center. The study was approved by the institutional review board of each of the participating centers.

Eligibility for TAVI was established after a multi-disciplinary approach as indicated by the current recommendations. The preoperative workout included MDCT scan to plan the most appropriate route of intervention and to establish the aortic size and dimensions. Aortic sizing and valve measurements were performed by the local team in each center. All centers adopted a transfemoral-first approach policy; other vascular accesses (trans-apical, trans-subclavian, etc.) were considered in cases in which the transfemoral access was not feasible. According to the local policy, TAVIs were performed under local or general anesthesia. The selection of prosthesis type and size was at the discretion of the treating physicians at each center. Pre-specified clinical and laboratory data were collected for all patients at baseline before the procedure, immediately after the procedure, during the index hospitalization, and during long-term follow-up. Collected data included medical history, electrocardiogram, echocardiography studies, MDCT measurements, laboratory tests, and clinical outcomes. Outcomes were collected according to the Valve Academic Research Consortium (VARC) 2 consensus document (7).

**Figure 1:**
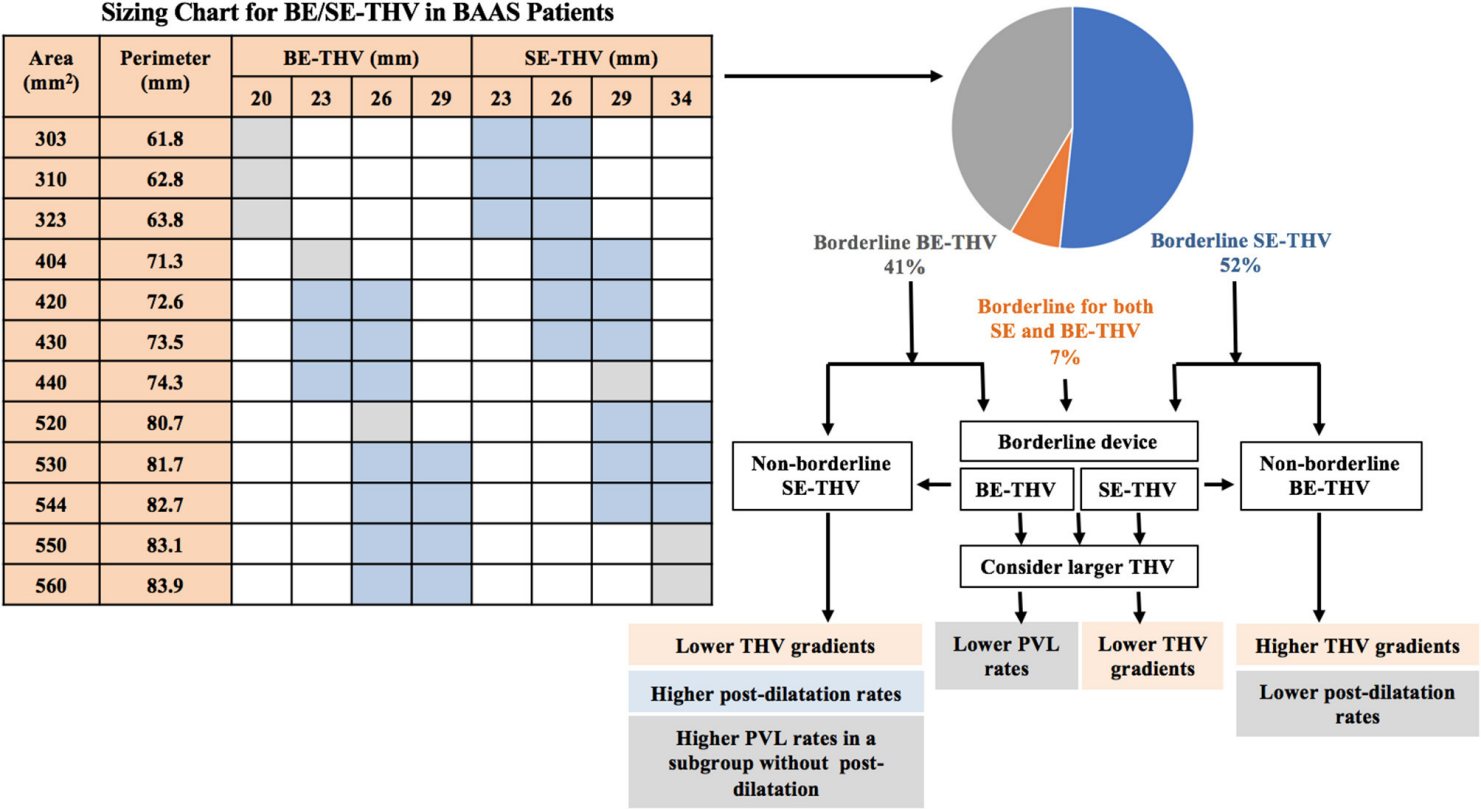
Valve selection in BAAS patients. BE, balloon-expandable; SE, self-expandable; THV, transcatheter heart valve; BAAS, borderline aortic annulus size; PVL, paravalvular leak.

BAAS was defined based on THV manufacturer sizing instructions; size cut-off ±1 mm for SE-THV (62.8 ± 1 mm for valve size of 23 vs. 26; 72.3 ± 1 for 26 vs. 29; 81.7 ± 1 for 29 vs. 34 mm) and borderline range for BE-THV (330–350 mm^2^ for valve size of 20 vs. 23 mm; 420–440 mm^2^ for 23 vs. 26 mm; 530–560 mm^2^ for 26 vs. 29 mm) (Figure 1) (8). Patients who underwent valve in valve or valve in ring procedures were excluded from the study.

### Study Devices

The Evolut R SE valve is constituted by a nitinol frame mounting three porcine pericardial leaflets. The valve is repositionable, partially recapturable, and it is deliverable using a dedicated delivery system 14/16-Fr compatible depending on valve size. The Evolut PRO device represents an evolution of its predecessor and features a porcine pericardial outer wrap that contributes to reduce the risk of residual PVL. Evolut R covers a wide range of sizes and is available in 23, 26, 29, and 34 mm sizes (9); the PRO valve is available in 23, 26, and 29 mm sizes (10).

The Sapien XT/3 BE valve incorporates a cobalt chromium stent that mounts bovine pericardial leaflets. Sapien 3, has both an inner and an outer polyethylene terephthalate fabric seal to minimize the risk of paravalvular leaks. The delivery system has an active 3-dimensional coaxial positioning catheter and a 16-Fr expandable sheath (11).

### Statistical Methods

Continuous variables were expressed as mean ± standard deviation and as median and interquartile range (IQR) and compared using Mann-Whitney test. Categorical variables were compared using Chi-square or Fisher's exact tests as needed. All analyses were conducted using Python version 3.5, *p* < 0.05 was considered statistically significant.

## Results

**Figure 2:**
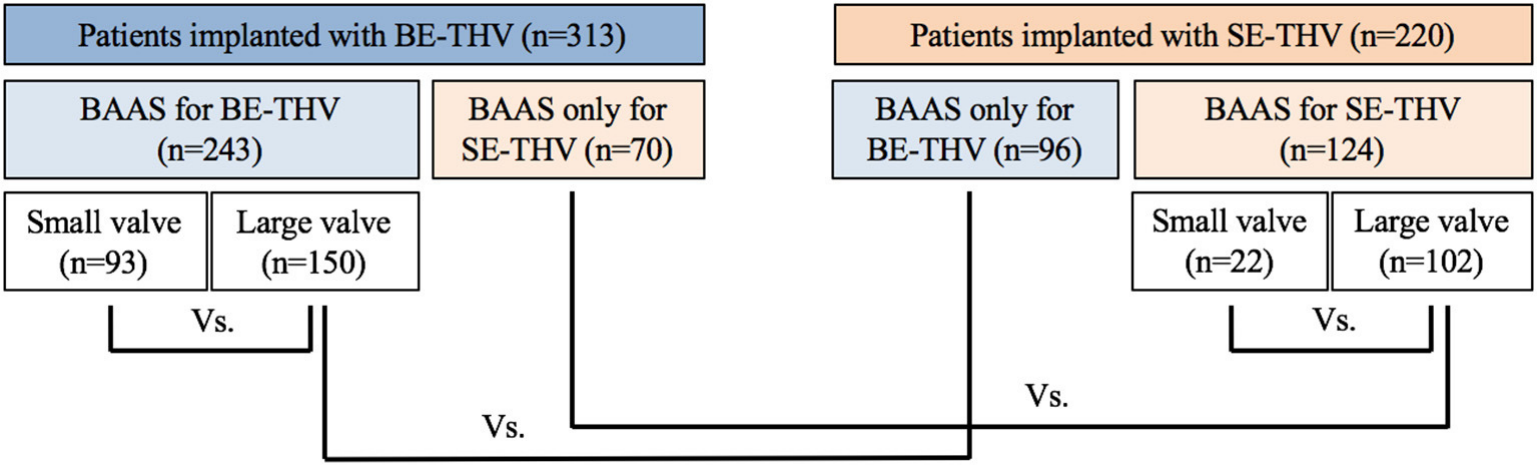
Flow chart for study cohort. BE, balloon-expandable; SE, self-expandable; THV, transcatheter heart valve, BAAS, borderline aortic annulus size.

Out of 2,352 patients following implantation of SE-THV (CoreValve, Evolut R and Evolut PRO) or BE-THV (Sapien XT, Sapien 3) with pre-procedural MDCT measurements, 124 were excluded due to valve in valve, valve in ring, or mitral valve interventions. Additional 38 patients with BAAS and an annulus area of 330–350 mm^2^ who were implanted with BE-THV were excluded from the analysis since the smaller valve size of 20 mm was not implanted. Eventually, 598 patients with BAAS as defined for at least one THV type, 309 for SE-THV, 248 for BE-THV and 41 patients for both devices were included in the analysis. Of them, 367 (61.4%) patients were implanted with borderline valves, while all others were implanted with non-borderline valves due to shift from SE-THV to BE-THV, or vice versa. The SE-THV group included 93 patients implanted with smaller valves, and 150 patients implanted with larger valves. In the BE-THV group, 22 patients were implanted with smaller valves, and 102 patients with larger valves (Figure 2).

In BAAS patients implanted with SE-THV, the baseline clinical characteristics of both groups (smaller and larger valves) did not differ, except for the New York Heart Association (NYHA) functional class (Table 1A). In addition, no significant differences were observed in imaging (echocardiography and MDCT) measurements (**Table 2A**). In BAAS patients implanted with BE-THV, differences were noted in left ventricular function (**Table 2B**). Other measured baseline clinical and imaging characteristics did not differ between smaller and larger valves implantation (Tables 1B, **2B**).

**Table 1A:** Baseline clinical characteristics of patients with BAAS for SE-THV, implanted with SE or non-borderline BE-THV.

	**Smaller borderline-SE-THV (*n* = 93)**	**Larger borderline-SE-THV (*n* = 150)**	**Non- borderline BE-THV (*n* = 70)**	*p*-value^*^	***p*-value^#^**
Female (%)	52 (56)	88 (58)	50 (71)	0.69	0.07
DM (%)	31 (33)	55 (36)	30 (42)	0.68	0.55
BMI kg/m^2^	27.6 ± 5.4 [27.3, 6.6]	27 ± 4.6 [26.7, 6.3]	27.9 ± 4.2 [27.3, 4.7]	0.21	0.09
STS score	4.2 ± 2.3 [3.79, 2.8]	4.3 ± 3.3 [3.45, 2.8]	4.48 ± 2.86 [4.18, 3.2]	0.29	0.28
PR interval (ms)	179.7 ± 40 [170, 49]	171 ± 44 [161, 34.5]	179 ± 38.7 [169, 24.5]	0.13	0.09
QRS interval (ms)	120 ± 33 [108, 52]	112 ± 35 [99, 35]	104 ± 33 [95, 45]	0.08	0.36
NYHA functional class:				0.03	0.38
I	0 (0)	2 (1.4)	1 (1.4)		
II	29 (33)	31 (22)	21 (31)		
III	51(58)	78 (55)	37 (54.4)		
IV	8 (9)	30 (21)	9 (13.2)		

*Values are mean ± SD or [median, interquartile range (IQR)] or n (%). DM, diabetes mellitus; BMI, body mass index; STS score, the society of thoracic surgeons scores; NYHA, New York Heart Association; SE, self-expandable; BE, balloon-expandable; THV, transcatheter heart valve*.

* *Smaller borderline-SE-THV vs. larger borderline-SE-THV*.

# *Larger borderline-SE-THV vs. non-borderline-BE*.

**Table 1B:** Baseline clinical characteristics of patients with BAAS for BE-THV, implanted with BE or non-borderline SE-THV.

	**Smaller borderline-BE-THV (*n* = 22)**	**Larger borderline-BE-THV (*n* = 102)**	**Non-borderline SE-THV (*n* = 96)**	*p*-value^*^	***p*-value^#^**
Female (%)	10 (45.4)	24 (23.5)	61 (63.5)	0.06	1.40e-08
DM (%)	10 (45.4)	43 (42.1)	43 (44.8)	1	0.24
BMI kg/m^2^	28.6 ± 3.7 [27.4, 4.6]	27.5 ± 4.4 [26.9, 5.4]	28 ± 4.9 [27.8, 6.9]	0.12	0.23
STS score	4 ± 4.5 [2.6, 2.1]	3.4 ± 2.25 [2.7, 2.1]	4.2 ± 2.4 [3.6, 2.6]	0.36	0.003
PR interval (ms)	185 ± 60 [189, 33.5]	181 ± 34 [182, 48.5]	174 ± 34.18 [171, 36.5]	0.42	0.12
QRS duration (ms)	135 ± 30 [148, 44]	121 ± 33 [107, 59]	106.9 ± 28 [100, 32]	0.11	0.05
NYHA functional class:				0.18	0.1
I	1 (4.5)	0 (0)	2 (2.2)		
II	5 (22.7)	22 (21.5)	29 (31.5)		
III	12 (54.5)	61 (59.8)	44 (47.8)		
IV	4 (18)	13 (12.7)	17 (18.5)		

*Values are mean ± SD or [median, interquartile range (IQR)] or n (%). DM, diabetes mellitus; BMI, body mass index; STS score, the society of thoracic surgeons scores; NYHA, New York Heart Association; SE, self-expandable; BE, balloon-expandable; THV, transcatheter heart valve*.

* *Smaller borderline-BE-THV vs. larger borderline-BE-THV*.

# *Larger borderline-BE-THV vs. non-borderline-SE*.

Baseline clinical and imaging characteristics of patients with borderline annulus for SE devices implanted with borderline large SE-THV or non-borderline BE-THV did not differ, except for aortic valve mean pressure gradient (Table 2A). Comparison between non-borderline SE-THV implantation to large BE-borderline valves implantation in patients with borderline annulus for BE devices showed more females and higher Society of Thoracic Surgeons (STS) score in patients implanted with non-borderline SE-THV compared to large BE-borderline valves (Table 1B). In addition, in patients implanted with non-borderline SE-THV the left main (LM) and right coronary artery (RCA) heights were shorter compared with patients implanted with larger BE-borderline valves (Table 2B).

**Table 2A:** Baseline echocardiography and MDCT characteristics of patients with BAAS for SE-THV implanted with SE or non-borderline BE-THV.

	**Smaller borderline-SE-THV (*n* = 93)**	**Larger borderline-SE-THV (*n* = 150)**	**Non- borderline BE-THV (*n* = 70)**	*p* value^*^	***p* value^**#**^**
Distance of LM (mm)	12.4 ± 3.2 [12, 4.1]	12.6 ± 2.6 [12.35, 3.6]	12.6 ± 2.7 [12, 2.5]	0.26	0.39
Distance of RCA (mm)	14.8 ± 3 [15, 3.9]	15.19 ± 2.9 [14.8, 3.7]	14.9 ± 2.8 [15, 4.1]	0.44	0.27
AVA cm^2^	0.65 ± 0.13	0.74 ± 0.17	0.7 ± 0.19	0.3	0.3
AV mean pressure (mmHg)	45 ± 15 [42, 21]	44 ± 14 [42, 21]	48 ± 12 [46, 16.5]	0.4	0.02
LV Function				0.189	0.52
Normal (>55%)	65 (70.6)	120 (82)	58 (84)		
Mild (45–54%)	7 (7.6)	8 (5.4)	6 (8.6)		
Mild-moderate (40–44%)	6 (6.5)	4 (2.7)	2 (2.8)		
Moderate (35–39%)	3 (3.2)	7 (4.7)	1 (1.4)		
Moderate-severe (30–34%)	5 (5.4)	3 (2)	2 (2.8)		
Severe (<29%)	6 (6.5)	4 (2.7)	0 (0)		

*Values are mean ± SD or [median, interquartile range (IQR)] or n (%). LM, left main; RCA, right coronary artery; AV, aortic valve; AVA, aortic valve area; SE, self-expandable; BE, balloon-expandable; THV, transcatheter heart valve; LV, left ventricular*.

* *Smaller borderline-SE-THV vs. larger borderline-SE-THV. #Larger borderline-SE-THV vs. non-borderline-BE*.

**Table 2B:** Baseline echocardiography and MDCT characteristics of patients with BAAS for BE-THV implanted with BE or non-borderline SE-THV.

	**Smaller borderline-BE-THV (*n* = 22)**	**Larger borderline-BE-THV (*n* = 102)**	**Non- borderline SE-THV (*n* = 96)**	*p*-value^*^	***p*-value^#^**
Distance of LM (mm)	13.8 ± 2.7 [14.1, 3.2]	13.69 ± 3 [13.5, 3.9]	12.45 ± 2.49 [12.2, 3.7]	0.36	0.0006
Distance of RCA (mm)	15.66 ± 3.2 [15, 3.7]	16 ± 3.6 [16, 4.6]	14.5 ± 3.12 [14, 4.9]	0.25	0.0003
AVA cm^2^	0.7 ± 0.16	0.76 ± 0.17	0.7 ± 0.19	0.55	0.144
AV mean pressure (mmHg)	45 ± 12.86 [48, 18]	41 ± 16 [41, 20.2]	44.2 ± 17.4 [40,21.5]	0.17	0.35
LV function				0.0192	0.35
Normal (>55%)	10 (47.6)	68 (70.8)	76 (80.8)		
Mild (45–54%)	3 (14.2)	14 (14.5)	12 (12.7)		
Mild-moderate (40–44%)	5 (23.8)	4 (4.1)	3 (3.2)		
Moderate (35–39%)	0 (0)	3 (3.1)	2 (2.1)		
Moderate-severe (30–34%)	1 (4.7)	5 (5.2)	1 (1)		
Severe (<29%)	2 (9.5)	2 (2)	0 (0)		

*Values are mean ± SD or [median, interquartile range (IQR)] or n (%). LM, left main; RCA, right coronary artery; AV, aortic valve; AVA, aortic valve area; SE, self-expandable; BE, balloon-expandable; THV, transcatheter heart valve; LV, left ventricular*.

* *Smaller borderline-BE-THV vs. larger borderline-BE-THV*.

# *Larger borderline-BE-THV vs. non-borderline-SE*.

**Figure 3:**
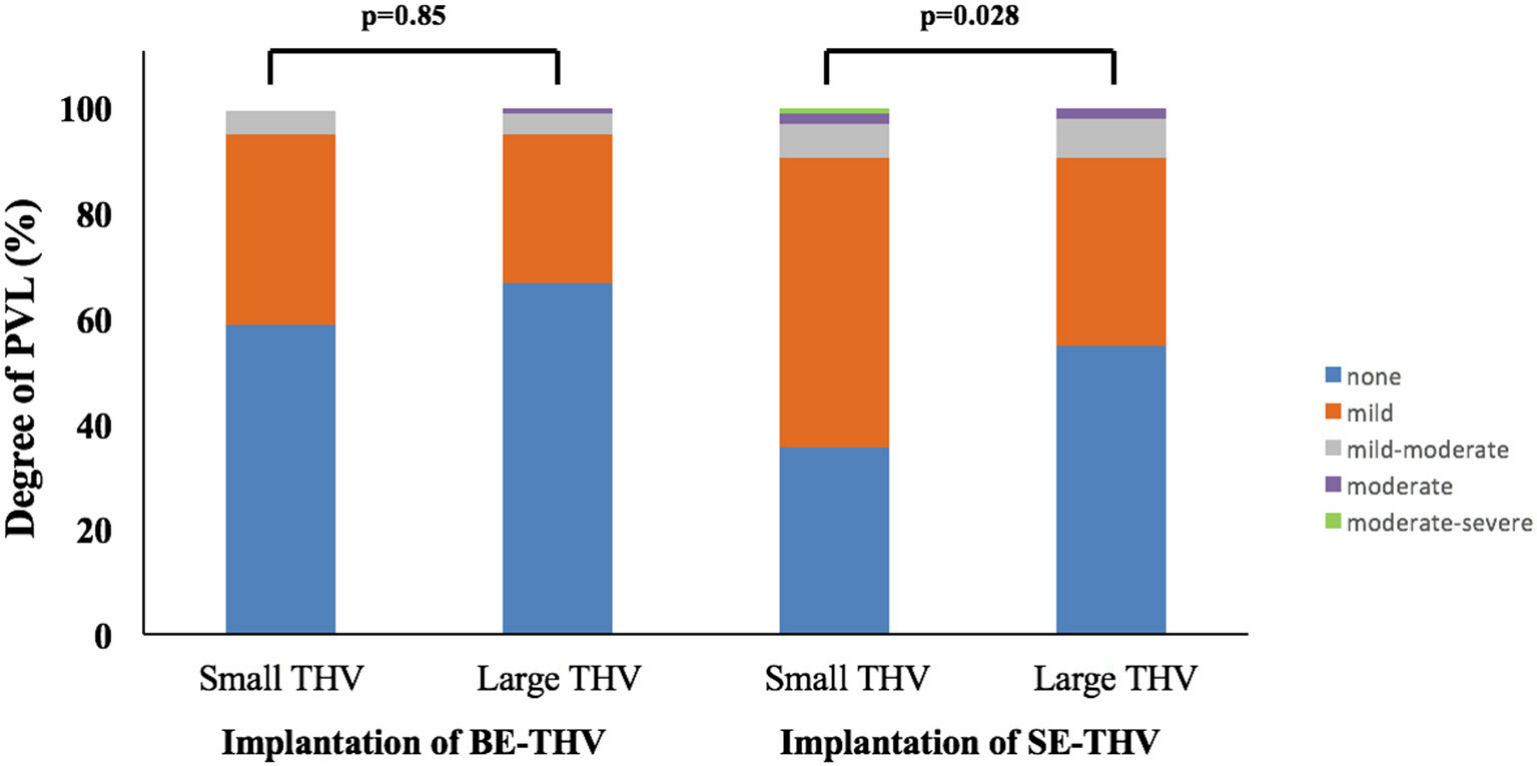
Incidence of paravalvular leak in patients with BAAS. BE, balloon-expandable; SE, self-expandable; THV, transcatheter heart valve; BAAS, borderline aortic annulus size; PVL, paravalvular leak.

**Figure 4:**
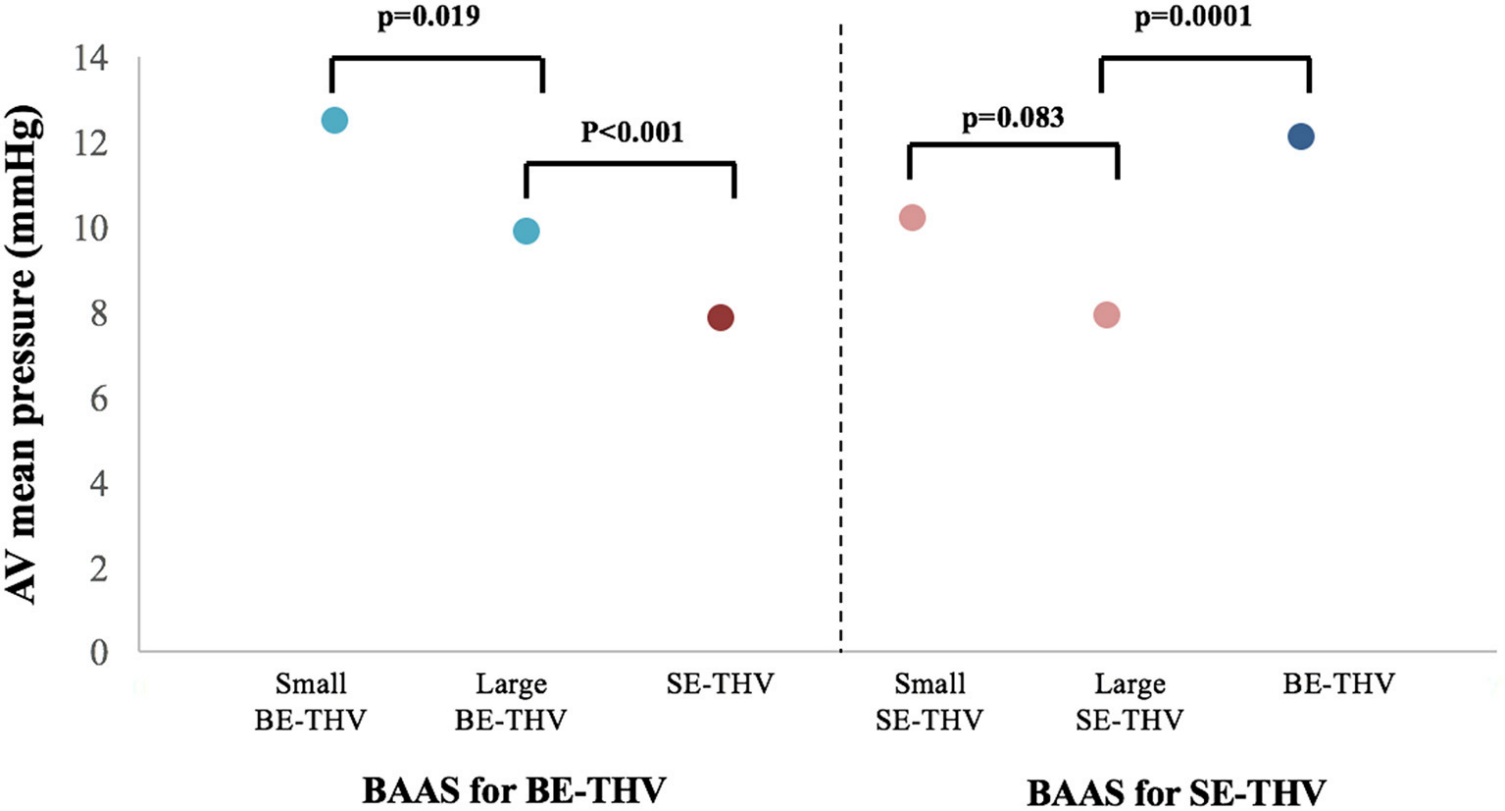
Transcatheter heart valve hemodynamics in patients with BAAS. AV, aortic valve; BE, balloon-expandable; SE, self-expandable; THV, transcatheter heart valve, BAAS, borderline aortic annulus size.

In the present cohort, favorable outcomes were observed while using larger valves in BAAS patients. For SE-THV, selection of larger valves compared to smaller valves was accompanied with significantly lower rates of PVL measured by both echocardiography (none: 54.6 vs. 35.5%, mild: 36 vs. 54.8%, mild to moderate: 7.3 vs. 6.4%, moderate: 2 vs. 2.1%, moderate to severe: 0 vs. 1%; pv = 0.0282; Figure 3; Table 3) and angiography (none: 85.3 vs. 68.8%, mild: 13.3 vs. 27.9%, moderate: 1.3 vs. 3.2%; pv = 0.0088; Table 3) and a trend toward lower gradients across the THV (7.9 ± 5.4 vs. 10.2 ± 10.8; pv = 0.083; Figure 4; Table 3); for BE-THV, selection of larger valves compared to smaller valves resulted in better hemodynamics with lower gradients across the THV (9.9 ± 3.7 vs. 12.5 ± 7.2; pv = 0.019; Figure 4; Table 3). In BE-THV no significant differences were demonstrated in PVL rates while comparing larger to smaller valves implantation in BAAS patients (Figure 2; Table 3). Selection of larger valves (either SE or BE) in BAAS patients did not change the rate of post-dilatation as well as adverse clinical outcomes such as new left bundle branch block (LBBB), rate of new pacemaker implantation, stroke or transient ischemic attack (TIA), annular rupture, coronary occlusion, or mortality (Table 3).

**Table 3:** Comparison of procedural and post-procedural outcomes between BAAS patients implanted with smaller vs. larger valves (SE or BE).

	**SE-borderline THV**		***p*-value**	**BE-borderline THV**		***p*-value**
	**Smaller (*n* = 93)**	**Larger (*n* = 150)**		**Smaller (*n* = 22)**	**Larger (*n* = 102)**	
Need for post-dilatation	37 (40)	57 (38)	0.786	1 (4.7)	8 (7.8)	1
device success (VARC-2) (%)	91 (97)	146 (97)	1	22 (100)	101(99)	1
Need for a second valve (%)	2 (2)	2 (1.3)	0.638	0 (0)	2 (1.9)	1
Valve malposition (%)	2 (2)	2 (1.3)	0.638	0 (0)	0 (0)	
Valve migration (%)	2 (2)	1 (0.6)	0.565	0 (0)	0 (0)	
Ischemic stroke/TIA (%)	3 (3.2)	2 (0.1)	0.616	2 (9)	0 (0)	0.12
Endocarditis (%)	2 (2)	0 (0)	1	0 (0)	1 (0.98)	1
Procedural mortality (%)	1 (1)	2 (0.1)	0.599	0 (0)	0 (0)	
Coronary obstruction (%)	0 (0)	0 (0)		0 (0)	0 (0)	
New LBBB (%)	21 (23)	34 (23)	0.882	4 (18.1)	29 (28)	0.471
New pacemaker (%)	16 (17)	17 (11.3)	0.4	2 (9)	21 (20)	0.521
AV mean pressure (mmHg)	10.2 ± 10.8 [8, 4.1]	7.9 ± 5.4 [7, 4]	0.083	12.5 ± 7.2 [12, 5.7]	9.9 ± 3.7 [9.6,5]	0.019
PVL per angiogram			0.0088			0.403
None	64 (68.8)	128 (85.3)		21 (95.4)	88 (86)	
Mild	26 (27.9)	20 (13.3)		1 (4.5)	14 (13.7)	
Moderate	3 (3.2)	2 (1.3)		0 (0)	0 (0)	
PVL per echo			0.028			0.856
None (%)	33 (35.5)	82 (54.6)		13 (59)	68 (66.6)	
Mild (%)	51 (54.8)	54 (36)		8 (36)	29 (28.4)	
Mild-mod (%)	6 (6.4)	11 (7.3)		1 (4.5)	4 (3.9)	
Moderate (%)	2 (2.1)	3 (2)		0 (0)	1 (0.98)	
Moderate-severe (%)	1 (1)	0 (0)		0 (0)	0 (0)	

*Values are mean ± SD or [median, interquartile range (IQR)] or n (%). VARC, valve academic research consortium; TIA, transient ischemic attack; LBBB, left bundle branch block; AV, aortic valve; PVL, paravalvular leak; echo, echocardiography; SE, self-expandable; BE, balloon-expandable; THV, transcatheter heart valve. Post-procedural outcomes (during index hospitalization) were endocarditis and new pacemaker implantation*.

Shift from large borderline SE-THV to non-borderline BE-THV was associated with higher gradients across the THV (7.98.5 ± 5.46.3 vs. 12.11 ± 4.53; pv <0.0001; Figure 4; Table 4); However, lower rates of post-dilatation were observed (38 vs. 12.8%; pv = 0.0001; Table 4), but without significant differences in PVL rates (Table 4). In a subgroup of patients who didn't undergo post-dilatation the PVL rates also did not differ (Table 4). Shift from large borderline BE-THV to non-borderline SE-THV resulted in lower gradients (9.9 ± 3.7 vs. 7.8 ± 3.5, pv <0.001; Figure 4; Table 4), and increased rates of post-dilatation (7.8 vs. 35.4%, pv <0.001; Table 4) with a trend toward increased overall PVL rated per echocardiography (33 vs. 45.8%, pv = 0.08; Table 4). In a subgroup of patients who didn't undergo post-dilation the PVL rates were increased in non-borderline SE-THV compared to large borderline BE-THV (none: 35.8 vs. 72%, mild: 51.2 vs. 25.8%, mild to moderate: 10.2 vs. 3.37%, moderate: 3 vs. 0%, pv = 0.001; Table 4).

**Table 4:** Comparison of procedural and post-procedural outcomes between patients implanted with large borderline-SE vs. non-borderline-BE devices; or large borderline-BE vs. non-borderline-SE devices.

	**BAAS only for SE device**		***p*-value**	**BAAS only for BE device**		***p*-value**
	**Large SE-borderline valve (*n* = 150)**	**BE- non-borderline valve (*n* = 70)**		**Large BE-borderline valve (*n* = 102)**	**SE- non-borderline valve (*n* = 96)**	
Need for post dilatation	57 (38)	9 (12.8)	0.0001	8 (7.8)	34 (35.4)	2.97e-06
Device success (VARC-2) (%)	146 (97)	70 (100)	0.554	101 (99)	93 (96.8)	0.11
Need for a second valve (%)	2 (1.3)	0 (0)	1	2 (1.9)	2 (2)	1
Valve malposition (%)	2 (1.3)	0 (0)	1	0 (0)	1 (1)	1
Valve migration (%)	1 (0.6)	0 (0)	1	0 (0)	0 (0)	
Ischemic stroke/TIA (%)	2 (0.1)	0 (0)	1	0 (0)	2 (2)	0.508
Endocarditis (%)	0 (0)	1 (1.4)	0.36	1 (0.98)	1 (1)	1
Procedural mortality (%)	2 (0.1)	0 (0)	0.528	0 (0)	1 (1.6)	0.927
Coronary obstruction (%)	0 (0)	2 (3.3)	0.166	0 (0)	1 (1.6)	0.927
New LBBB (%)	34 (23)	17 (28.3)	0.615	29 (28)	27 (28.1)	0.737
New pacemaker (%)	17 (11.3)	9 (15)	0.579	21 (20)	17 (17.7)	0.499
AV mean pressure (mmHg)	7.9 ± 5.4 (7, 4)	12.1 ± 4.5 (12, 8)	1.70e-10	9.9 ± 3.7 [9.6, 5]	7.8 ± 3.5 (7, 5)	3.90e-05
PVL per echo^+^			0.246			0.191
None (%)	82 (54.6)	47 (67.1)		68 (66.6)	52 (54)	
Mild (%)	54 (36)	20 (28.5)		29 (28.4)	36 (37.5)	
Mild-mod (%)	11 (7.3)	3 (4.3)		4 (3.9)	6 (6.25)	
Moderate (%)	3 (2)	0 (0)		1 (0.98)	2 (2)	
Moderate-severe (%)	0 (0)	0 (0)		0 (0)	0 (0)	
Overall PVL per echo^+^	68 (45.3)	23 (32.8)	0.106	34 (33)	44 (45.8)	0.0818
	**Large SE-borderline** **valve** **(*****n*** **=** **93)**	**BE- non-borderline** **valve** **(*****n*** **=** **32)**		**Large BE-borderline** **valve** **(*****n*** **=** **94)**	**SE- non-borderline** **valve** **(*****n*** **=** **39)**	
PVL per echo^+^			0.699			0.0015
None (%)	42 (45.1)	16 (50)		68 (72.3)	14 (35.8)	
Mild (%)	40 (45.9)	14 (43.7)		23 (25.8)	20 (51.2)	
Mild-moderate (%)	10 (11.4)	2 (6.25)		3 (3.37)	4 (10.2)	
Moderate (%)	1 (1.1)	0 (0)		0 (0)	1 (3)	
Moderate-severe (%)	0 (0)	0 (0)		0 (0)	0 (0)	
Overall PVL per echo^+^	51	16	0.413	26	25	0.0003

*Values are mean ± SD or [median, interquartile range (IQR)] or n (%). VARC, valve academic research consortium; TIA, transient ischemic attack; LBBB, left bundle branch block; AV, aortic valve; PVL, paravalvular leak; SE, self-expandable; BE, balloon-expandable; THV, transcatheter heart valve; echo, echocardiography*.

+ *subgroup of patients who did undergo post-dilatation. Post-procedural outcomes (during index hospitalization) were endocarditis and new pacemaker implantation*.

## Discussion

Borderline annulus size (annular dimensions in between 2 valve sizes) is common among patients undergoing TAVI, however, the most effective THV selection strategy for these patients remains unclear. The present study of 598 patients with severe symptomatic AS undergoing TAVI based on the ISRAELI-TAVI registry is the largest observational study to date comparing clinical outcomes according to size selection of SE-THV and BE-THV in patients with BAAS. The main findings of our study (Figure 1) are as follows:

- In patients with BAAS, the larger THV device reduced PVL rates and optimized THV hemodynamics.
- Selection of a larger valve in BAAS patients did not increase adverse clinical outcomes such as new LBBB, rate of new pacemaker implantation, stroke or TIA, annular rupture, coronary occlusion or mortality.
- Shift from borderline THV to non-borderline THV modified THV hemodynamics and post dilatation rates. Shift from borderline SE-THV to non-borderline BE-THV was associated with lower post dilatation rates, but with higher gradients. Shift from borderline BE-THV to non-borderline SE-THV led to optimized THV hemodynamics, but with increased post-dilatation rates; In addition, in a subgroup of patients in whom post-dilatation was not performed, increased PVL rates were observed.

Large size THV implantation was previously shown to be associated with favorable hemodynamics and lower PVL rate, both are important determinants of clinical outcomes in AS patients following TAVI (4, 12). In fact, evidence shows deleterious prognostic effects even with mild residual PVL after TAVI, including increased mortality (13, 14). In addition, higher post-TAVI transaortic gradients are associated with decreased THV long-term durability (15). The advantages of implanting larger, over smaller, devices were indeed reflected in our cohort of BAAS patients by reduced PVL rates and optimized THV hemodynamics. These findings are particularly important in the current era, in which younger and relatively healthier patients are being treated with TAVI and in whom the durability of the device is extremely important to minimize the need for future reintervention. Importantly, the use of a larger THV in BAAS patients was not associated with increased adverse outcomes commonly encountered with large prostheses, such as conduction disturbances, annular rupture and coronary occlusion (16, 17). Given the above results, the present study strengthens recent results from the PARTNER 3 trial (6) and advocates the selection of a large THV for BAAS patients undergoing TAVI with either SE or BE prostheses.

In our cohort of BAAS patients, 93% of cases were defined as BAAS for one device only (i.e., either BE or SE). In these patients, it is thus conceivable to apply a strategy of selecting the non-borderline device whenever possible. In fact, non-borderline devices were selected over borderline devices in 38.6% of patients in our cohort. We found that selection of non-borderline SE-THV over borderline BE-THV led to lower gradients. On the other hand, selection of non-borderline BE-THV over borderline SE-THV was associated with lower rates of post-dilatation, but at the cost of increased gradients. This trade-off between PVL and higher gradients was repeatedly described in comparative studies between BE and SE devices both in tricuspid and bicuspid AS patients (18, 19). These changes were mainly attributed to THV mechanical characteristics, such as annular/supra-annular valve position, radial forces, and the presence of outer skirt (15). Therefore, our findings point out that similar considerations taken while selecting THV type for non-BAAS patients (including calcifications, coronary height, sinus of valsalva dimensions), should be applied also in BAAS patients.

We acknowledge several limitations of our study. The main limitation is the observational nature of the study. Therefore, undocumented factors, such as sinus of valsalva diameter or calcium score, may have affected device selection. In addition, potential impact of unknown or unmeasured confounding factors on study outcomes cannot be excluded. The low number of patients implanted with smaller BE-valve may affect the significance of the results and even necessitated the exclusion of patients with annulus measurements of 330–350 mm^2^ from the analysis. BE-TVH over or under-sizing by over or under-inflation of the valve balloon in order to fine tune the valve dimensions was not registered and might have affected *in-situ* valve size. Nevertheless, the practice of over/under-inflation in the four centers was according to a known algorithm proposed by the company.

The results of the present study support, for both devices (BE and SE), the selection of larger valves for TAVI candidates with BAAS. Shifting from borderline devices to non-borderline devices resulted in significant changes in post-dilatation, PVL, and gradients across the THV. Therefore, our findings point out that the same consideration taken while selecting THV type for non-BAAS patients, should be applied in BAAS patients, and whenever a borderline device is selected the larger valve device should be recommended.

## Conclusions

The results of the present study support, for both devices (BE and SE), the selection of larger valves for TAVI candidates with BAAS. Shifting from borderline devices to non-borderline devices resulted in significant changes in PVL and THV hemodynamics. Therefore, our findings point out that the same consideration taken while selecting THV type for non-BAAS patients, should be applied in BAAS patients, and whenever a borderline device is selected the larger valve device is recommended.

## Data Availability Statement

The original contributions presented in the study are included in the article/supplementary materials, further inquiries can be directed to the corresponding author.

## Author Contributions

YT-B, RK, JB-S, and PC conceived the project, designed and interpreted the results, and wrote the manuscript. NB conducted all analyses and interpreted the results. HV-A, AH, BK, HD, GP, MK, AF, ASt, IM, ASe, and AB coordinated and designed data collection and interpreted the results. All authors contributed to the article and approved the submitted version.

## Conflict of Interest

The authors declare that the research was conducted in the absence of any commercial or financial relationships that could be construed as a potential conflict of interest.

## Publisher's Note

All claims expressed in this article are solely those of the authors and do not necessarily represent those of their affiliated organizations, or those of the publisher, the editors and the reviewers. Any product that may be evaluated in this article, or claim that may be made by its manufacturer, is not guaranteed or endorsed by the publisher.
